# GABAergic dysfunction in postmortem dorsolateral prefrontal cortex: implications for cognitive deficits in schizophrenia and affective disorders

**DOI:** 10.3389/fncel.2024.1440834

**Published:** 2024-09-24

**Authors:** Hannah Hughes, Lillian J. Brady, Kirsten E. Schoonover

**Affiliations:** ^1^Graduate Biomedical Sciences Program, School of Medicine, University of Alabama at Birmingham, Tuskegee, AL, United States; ^2^Department of Psychiatry, School of Medicine, University of Alabama at Birmingham, Tuskegee, AL, United States; ^3^Comprehensive Neuroscience Center, University of Alabama at Birmingham, Tuskegee, AL, United States; ^4^Department of Psychology and Sociology, College of Arts and Sciences, Tuskegee University, Tuskegee, AL, United States

**Keywords:** bipolar disorder, gamma oscillations, interneuron, major depressive disorder, working memory, sex differences

## Abstract

The microcircuitry within superficial layers of the dorsolateral prefrontal cortex (DLPFC), composed of excitatory pyramidal neurons and inhibitory GABAergic interneurons, has been suggested as the neural substrate of working memory performance. In schizophrenia, working memory impairments are thought to result from alterations of microcircuitry within the DLPFC. GABAergic interneurons, in particular, are crucially involved in synchronizing neural activity at gamma frequency, the power of which increases with working memory load. Alterations of GABAergic interneurons, particularly parvalbumin (PV) and somatostatin (SST) subtypes, are frequently observed in schizophrenia. Abnormalities of GABAergic neurotransmission, such as deficiencies in the 67 kDA isoform of GABA synthesis enzyme (GAD67), vesicular GABA transporter (vGAT), and GABA reuptake transporter 1 (GAT1) in presynaptic boutons, as well as postsynaptic alterations in GABA_*A*_ receptor subunits further contribute to impaired inhibition. This review explores GABAergic abnormalities of the postmortem DLPFC in schizophrenia, with a focus on the roles of interneuron subtypes involved in cognition, and GABAergic neurotransmission within presynaptic boutons and postsynaptic alterations. Where available, comparisons between schizophrenia and affective disorders that share cognitive pathology such as bipolar disorder and major depressive disorder will be made. Challenges in directly measuring GABA levels are addressed, emphasizing the need for innovative techniques. Understanding GABAergic abnormalities and their implications for neural circuit dysfunction in schizophrenia is crucial for developing targeted therapies.

## Introduction to GABA abnormalities in schizophrenia

**FIGURE 1:**
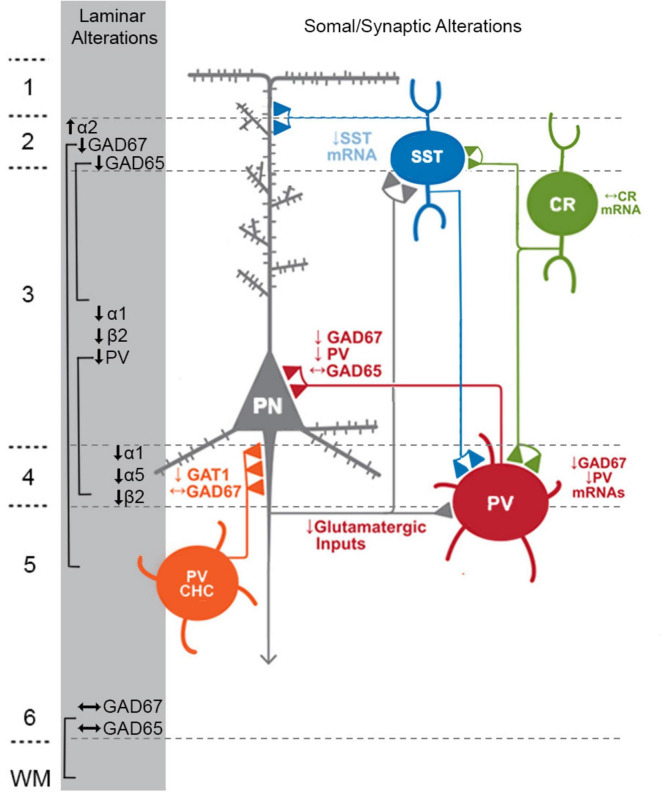
Schematic of components with consistent findings in schizophrenia of the DLPFC microcircuitry thought to be responsible for gamma oscillations and working memory. In the unaffected circuit, gamma oscillations are generated in supragranular layers via a microcircuit formed by connections between layer 3 pyramidal neurons (PNs), somatostatin neurons (SSTs), and parvalbumin-basket cells (PVBCs); SSTs dampen the excitation of L3PNs at their apical dendrites and also project inhibitory connections onto the cell body of PVBCs; the spiking of PVBCs synchronizes the firing of neighboring PNs at gamma frequency. Furthermore, SSTs project inhibitory connections onto PVBCs, regulating their spiking. In addition, the persistent firing of PNs during WM tasks arises, in part, from recurrent excitation among them and is shaped by dendritic inhibition and perisomatic inhibition thought to arise from SSTs and PVBCs, respectively. Calretinin (CR)-expressing interneurons target both PV and SST neurons, serving to disinhibit pyramidal neurons. PV chandelier cells (PV CHC) form connections directly onto the axon initial segment, and thus are poised to rapidly suppress the output of PNs. In schizophrenia, alterations of key transcripts by laminae are shown on the left. Lower PV and GAD67 in the PVBC terminals suggests that both presynaptic inhibition of PVBCs onto PNs is lower. Although the cell type-specific deficits in SST neurons are less well-defined, lower SST mRNA expression suggests these neurons may have an impaired ability to laterally inhibit other populations of PNs. CR cells do not exhibit lower levels of CR mRNA or protein in the illness, suggesting that these cells may not be affected in schizophrenia. PV CHCs express normal levels of GAD67, but lower GAT1 in their cartridges and higher levels of the GABAA receptor α2 subunit on the axon initial segment of the PN. Together, these alterations suggest that GABA signaling from PV CHCs might be higher in schizophrenia.

Schizophrenia is a debilitating and complex neuropsychiatric disorder characterized by an altered perception of reality. The symptoms of schizophrenia have been divided into three subtypes: positive (hallucinations and delusions), negative (lack of emotion and social withdrawal), and cognitive (learning, attention, and memory deficits), with cognitive symptoms being indicative of a more severe prognosis (Green, 1996; Harvey et al., 1998). Cognitive deficits in schizophrenia are hypothesized to be caused by neural circuit dysfunction involving imbalances between excitatory and inhibitory synaptic transmission (Weinberger et al., 1986; Carter et al., 1998; Glahn et al., 2005; Van Snellenberg et al., 2006; Minzenberg et al., 2009; Buffalo et al., 2011; Michalareas et al., 2016; Bastos et al., 2018). Inhibitory interneurons release the inhibitory neurotransmitter GABA, tuning the firing frequency of local pyramidal neurons that release the excitatory neurotransmitter glutamate and, in turn, excite the interneurons to maintain appropriate circuit and network function (Figure 1) (Goldman-Rakic, 1995; Wang et al., 2013; Gonzalez-Burgos et al., 2015; Chen et al., 2017). In schizophrenia, GABAergic interneurons are thought to underlie disruptions in neuronal synchrony contributing to observed cognitive deficits (Gonzalez-Burgos and Lewis, 2008; Lewis and Gonzalez-Burgos, 2008; Jones, 2010; Gandal et al., 2012). Thus, alterations of specific subtypes of GABAergic interneurons contributing to imbalanced neural circuitry are thought to ultimately result in cognitive impairment in schizophrenia (Nguyen et al., 2014).

Thus, it is crucial to investigate how abnormalities of the GABA system contribute to cognitive deficits in schizophrenia. Furthermore, understanding the fundamental mechanisms governing GABAergic synaptic transmission will contribute to the creation of evidence-based interventions for other disorders that exhibit GABAergic dysfunction. Indeed, alterations of GABAergic signaling have also been associated with bipolar disorder (BPD) and major depressive disorder (MDD), leading to speculation that these disorders could exhibit a shared pathology that results in the cognitive impairments present in all three disorders (Barch, 2009; Reichenberg et al., 2009).

Therefore, here we focus on GABAergic abnormalities of the human postmortem dorsolateral prefrontal cortex (DLPFC) believed to be the neural substrate for cognitive and working memory deficits in hopes of providing insight for the development of better comorbid treatments beyond antipsychotics and antidepressants. We highlight the literature outlining DLPFC GABAergic abnormalities in schizophrenia and discuss gaps in the existing literature that preclude our understanding of how these deficits and subsequent circuit dysfunctions manifest.

## Oscillation coherence in working memory deficits associated with schizophrenia

Many of the GABAergic neurons (80–90%) in the primate prefrontal cortex (PFC) consist of unique, non-overlapping subtypes based on the expression of one of three calcium-binding proteins—calbindin (CB; ∼20%), calretinin (CR; ∼45%), or parvalbumin (PV; ∼20%); (Conde et al., 1994; Gabbott and Bacon, 1996; del Río and DeFelipe, 1997a; Barinka and Druga, 2010). Additionally, each of the calcium-binding protein interneuron populations often co-express one of four neuropeptides— neuropeptide Y (NPY), vasoactive intestinal polypeptide (VIP), somatostatin (SST), and cholecystokinin (CCK). Indeed, CB-positive cells are often also SST-positive (Ang and Shul, 1995; Gonzalez-Albo et al., 2001; Suarez-Sola et al., 2009; Zaitsev et al., 2009), and CR-positive cells are often VIP-positive (Gabbott and Bacon, 1997). These interneurons contribute heavily to oscillations that synchronize circuit activity in the DLPFC and other brain regions (Dienel and Lewis, 2019). The synchronization of neuronal activity by oscillations is an innate mechanism that occurs to assist in proper cognitive function (Colgin and Moser, 2010) and functional connectivity between brain regions (Popescu et al., 2009).

Neuronal oscillations in the delta (0.5–3.5 Hz), theta (3.5–7 Hz), alpha (8–13 Hz), beta (18–25 Hz), and gamma (30–80 Hz) frequency ranges have distinctive roles in cognitive function. Slow-wave deep sleep and interconnected neuronal network connections at longer ranges are associated with delta oscillations (Harmony, 2013; Hiltunen et al., 2014), while rapid eye movement and shallow sleep as well as memory encoding involving the hippocampus are connected to theta oscillations (Cantero et al., 2003; Zielinski et al., 2020). When the brain is active yet relaxed or at rest, alpha waves are involved (Klimesch, 2012; Xavier et al., 2020), and awakened states, conscious thoughts, logical thinking, and attention are linked to beta oscillations (Espenhahn et al., 2019; Schmidt et al., 2019). These lower frequency oscillations can couple with each other to alter interneuron and pyramidal cell activity in cortical regions leading to changes in cognitive function altered in schizophrenia.

The coupling of oscillations between frequencies is referred to as neuronal coherence and is a fundamental feature of oscillatory activity across brain regions and within neural networks (Varela et al., 2001). Impaired coupling between frequencies where one or more oscillation ranges are altered has been linked to increased schizophrenia risk and susceptibility. For example, cross-frequency coupling between the theta and beta ranges are associated with working memory (WM) (Bygrave et al., 2019), and deficits in this coherence where theta is elevated, while beta is reduced can result in enhanced risk for psychosis associated with schizophrenia (Cousijn et al., 2015). Additionally, impaired alpha/beta coupling has been implicated during the consolidation period of WM in patients with schizophrenia compared with unaffected patients (Erickson et al., 2017). Furthermore, EEG recordings in individuals with schizophrenia that assessed delta/alpha oscillatory coherence have been shown to underlie physiological mechanisms associated with psychosis and could be associated with dysfunction of thalamocortical connectivity (Howells et al., 2018). Finally, several groups have provided evidence of coupling between theta and gamma oscillations, referred to as theta-gamma coupling (TGC), that underlies WM processes associated with schizophrenia (Engel et al., 2001; Canolty and Knight, 2010). GABAergic interneurons, particularly inhibition onto PV-positive ones, play a distinct role in regulating theta-gamma interactions (Wulff et al., 2009). In all, coherence between and within oscillation ranges impacts working memory in a variety of ways, thereby increasing susceptibility to schizophrenia.

## Gamma oscillations and GABA neuron physiology

A large portion of research into WM impairments in schizophrenia, especially work completed in human postmortem tissue, has focused on the neural substrate of high-frequency gamma oscillations (30–80 Hz), as these oscillations are important for higher brain processes such as learning, memory, and cognition (Andersson et al., 2012). For gamma oscillations to generate, pyramidal neurons fire asynchronously leading to depolarization of GABAergic interneurons which ultimately causes feedback inhibition of connected principal cells (Dienel and Lewis, 2019). This cycle of asynchronous firing and feedback inhibition results in coordinated firing of pyramidal cells at a frequency within the gamma range (Constantinidis et al., 2002; Gonzalez-Burgos et al., 2015; Bastos et al., 2018). The power of gamma oscillations within the DLPFC are lower in individuals with schizophrenia (Uhlhaas and Singer, 2010; Dienel and Lewis, 2019), and fail to increase in power during a WM task (Cho et al., 2006), in contrast to that observed in unaffected comparison individuals (UCs).

Abnormalities of the GABAergic interneurons and excitatory pyramidal neurons within the superficial layers of the DLPFC are believed to be the neural substrate of altered gamma power and WM impairments in schizophrenia (Lewis et al., 1999; Lewis and Moghaddam, 2006; Lewis and Hashimoto, 2007), as well as other comorbid psychiatric disorders like BPD (Benes and Berretta, 2001; Knable et al., 2004; Benes et al., 2008) and MDD (Luscher et al., 2011). Many studies of schizophrenia have been dedicated to abnormalities of the GABAergic system (Uehara et al., 2015), with a focus on the specific alterations of the genetic expression and function of enzymes and proteins expressed in GABAergic interneurons and involved in inhibitory synaptic transmission (Howes and Shatalina, 2022). Indeed, schizophrenia genome-wide association studies (GWAS) have identified the gene *SLC32A1*, which encodes the vesicular GABA transporter (vGAT) and is involved in controlling the vesicular uptake of GABA (Ripke et al., 2011; Ikeda et al., 2019; Howes and Shatalina, 2022). Therefore, postmortem reports of alterations in GABAergic neurotransmission are of key importance to understanding schizophrenia pathology, and may also contribute to the shared pathology within affective disorders such as BPD and MDD.

## Alterations of parvalbumin interneurons in schizophrenia DLPFC

PV interneurons are predominantly localized to layers 3–4 within the DLPFC (Chung et al., 2016b; Dienel et al., 2021), and compose approximately 25% of all cortical interneurons (Conde et al., 1994). PV cells can be divided into two different subtypes, basket cells (PVBCs) and chandelier cells (ChCs). PVBCs form synapses onto the somal bodies, proximal dendritic shafts, and spines of local layer 3 pyramidal neurons (Williams et al., 1992; Melchitzky et al., 1999; Zaitsev et al., 2009), whereas ChCs target the axon initial segment of pyramidal neurons (Somogyi, 1977; Lewis and Lund, 1990; Williams et al., 1992; Lund and Lewis, 1993; Melchitzky and Lewis, 2003). Although the unique populations of PV interneurons can be readily identified, methodological and technical limitations still exist that prohibit a clear distinction between PVBCs and ChCs. Therefore, for the purpose of this review, we will not distinguish between the PV interneuron subtypes unless otherwise specified.

Alterations of PV interneurons in the DLPFC of individuals with schizophrenia have been consistently reported (Hashimoto et al., 2003; Fung et al., 2010; Chung et al., 2016b; Volk et al., 2016; Enwright et al., 2018; Tsubomoto et al., 2019). Lower levels of DLPFC PV mRNA have been observed in total gray matter homogenates, layers 3–4, and within individual layer 4 PV neurons (Hashimoto et al., 2003; Dienel et al., 2023b). Such deficits in DLPFC PV mRNA translate to reductions in PV protein levels in cell bodies (Chung et al., 2016a) and axon terminals localized within the DLPFC (Glausier et al., 2014), without a reduction of PV-positive neurons (Woo et al., 1997; Hashimoto et al., 2003; Tooney and Chahl, 2004; Chung et al., 2016a; Enwright et al., 2016). At the level of the synapse, DLPFC PV-positive axon terminals exhibit lower levels of GAD67 (Curley et al., 2011) and PV protein levels (Glausier et al., 2014).

However, these alterations may be specific to PV interneuron subtype. Indeed, GAD67 protein expression is unaltered in the axon cartridges of ChCs within the prefrontal cortex (Rocco et al., 2016a). Although ChC cartridges do not appear to express alterations in GABA synthesis, the density of ChC cell cartridges identified by GABA reuptake transporter 1 (GAT1) mRNA has been reported to be lower in DLPFC layers 2–4 in individuals with schizophrenia (Woo et al., 1998; Pierri et al., 1999), although it is uncertain if these findings reflect a methodological limitation of detectability. Indeed, the density of cartridges identified by vGAT is unaltered in the illness (Rocco et al., 2017).

Fewer studies have been conducted examining PV interneurons within the PFC of individuals diagnosed with BPD or MDD. In total gray matter homogenate, Chung et al. (2016b) reported significantly lower levels of DLPFC PV mRNA in both BPD and MDD subjects compared to UC subjects, which were also simultaneously significantly higher than those diagnosed with schizophrenia. In contrast, utilizing whole tissue homogenate of the DLPFC, Sibille et al. (2011) observed a deficit only in those diagnosed with BPD. Although such differences could be attributed to cohort or tissue sampling differences, further testing is necessary to determine if distinct alterations can be differentially attributed to specific mood disorders.

## Lack of schizophrenia-associated calretinin interneuron alterations

In primate DLPFC, CR interneurons represent approximately 50% of all DLPFC GABA neurons (Conde et al., 1994). Although the somata and dendritic and axonal processes are found in all cortical layers, the cell bodies of this interneuron subtype are most prevalent in layers 1–superficial 3 (Conde et al., 1994; Daviss and Lewis, 1995), and these observations are supported by findings of significantly higher levels of CR mRNA in layer 2 in comparison to layer 4 (Chung et al., 2016b). However, multiple types of CR-positive cells are present within each layer (Gabbott et al., 1997). Indeed, bipolar CR-positive cells within human PFC often co-express VIP (Gabbott and Bacon, 1997). Despite the high proportion of CR neurons to all GABAergic interneuron subtypes (Conde et al., 1994), CR axonal boutons comprise only about 16% of all GABAergic boutons in the DLPFC, but are present in all cortical layers (Rocco et al., 2016b). These boutons primarily target dendrites of PV (Donato et al., 2013; Pi et al., 2013) and SST neurons (Pfeffer et al., 2013; Pi et al., 2013), contributing to the spatial tuning of local microcircuits.

In schizophrenia PFC, no alterations of CR mRNA or protein have been observed (Hashimoto et al., 2003; Hashimoto et al., 2008; Chung et al., 2016b). However, these alterations may vary by cohort, brain region, or cortical layer: Volk et al. (2012) observed a cohort subset effect of a 9% upregulation in DLPFC of individuals with schizophrenia that did not exhibit significant downregulations in other inhibitory markers. Furthermore, Tsubomoto et al. (2019) observed a significant 41% upregulation of CR mRNA in primary visual cortex (V1) but not DLPFC, although the cohort was not subclustered in the same manner as Volk et al. (2012). Irrespective of CR mRNA or protein levels, studies of CR-immunoreactive somata have found no difference in CR cell density and soma size across all layers of the DLPFC (Daviss and Lewis, 1995; Reynolds and Beasley, 2001; Reynolds et al., 2001; Beasley et al., 2002; Reynolds et al., 2002; Hashimoto et al., 2003; Tooney and Chahl, 2004; Oh et al., 2012), suggesting that the identification of CR cells may not be affected by methodological limitations of detection thresholds like other interneuron types (e.g., SST cells, see below).

Even with the growing abundance of studies focused on CR cells in schizophrenia, there are limited and variable investigations of CR cells within postmortem cortical regions in individuals with mood disorders, such as BPD or MDD. A study of CR interneuronal density within the anterior cingulate cortex revealed no alterations in BPD subjects (Cotter et al., 2002), but a later study examining the density of CR neurons within layer 2 of the DLPFC of individuals with BPD revealed a higher density of large CR-immunoreactive cells (Sakai et al., 2008). Reports of CR interneuron density in MDD DLPFC have been varied (Beasley et al., 2002; Reynolds et al., 2002; Oh et al., 2012), but suggest a CR neuronal density deficit specifically in DLPFC layer 1 in MDD (Oh et al., 2012). However, a separate investigation of postmortem auditory cortex revealed a lower density of CR-positive cells without respect to cortical layer in individuals with MDD (Smiley et al., 2016). Thus, perhaps as in schizophrenia, CR alterations (or lack thereof) may differ by cortical region and layer in BPD and MDD.

## Consistent and robust deficiencies of somatostatin neurons in schizophrenia

In the primate DLPFC, SST neurons account for approximately 30% of all interneurons (Rudy et al., 2011) and are found in the highest density within layer 2 (Lewis et al., 1986; Gabbott and Bacon, 1996; Dienel et al., 2021). SST interneurons can be further divided into unique subtypes. Those located within the superficial layers of the cortex often co-express calcium-binding protein CB (Gonzalez-Albo et al., 2001; Zaitsev et al., 2009) and primarily target the distal dendrites of local pyramidal neurons (Melchitzky and Lewis, 2008), temporally tuning the oscillations associated with WM capability (Abbas et al., 2018; Torres-Gomez et al., 2020; Froudist-Walsh et al., 2021). However, those within deep layer 6 and the superficial white matter typically lack CB mRNA (Ang and Shul, 1995; Suarez-Sola et al., 2009) and form long-range axonal projections (Tomioka and Rockland, 2007).

Due to their role in WM capability, SST neurons within superficial layers but not the deep layers of the cortex have been predominantly studied in schizophrenia. Therefore, here we focus on the findings of SST interneurons within cortical layers 1–3.

Downregulation of SST mRNA is perhaps the most highly replicated finding in schizophrenia. Indeed, lower levels of SST mRNA have been observed across multiple methodologies, subject cohorts, cortical regions, and laminae (Melchitzky and Lewis, 2008; Morris et al., 2008; Fung et al., 2010; Tsubomoto et al., 2019; Dienel et al., 2023a; Dienel et al., 2023b). Although a reduction of SST-positive interneuron density in schizophrenia has been previously reported (Morris et al., 2008), recent work illustrates that such findings were a result of a limited detection threshold of the methodologies available at the time; newer approaches with a higher level of detection sensitivity indicate a significant downregulation of SST mRNA per neuron without a loss of SST interneuron density in the illness (Dienel et al., 2023b).

In contrast to the consistency of findings in schizophrenia, findings of PFC SST levels in BPD or MDD differ substantially across studies. Individuals with MDD, but not BPD, exhibit lower SST mRNA levels (Sibille et al., 2011; Dienel et al., 2023a), but this data is contrasted by the work of Fung et al. (2014) who reported robust deficits of SST mRNA in BPD. However, a singular report investigating SST mRNA in deep layer 6/superficial white matter found no alterations within MDD or BPD (Dienel et al., 2023a), suggesting that there may be differential effects of both disease and cortical layer.

## Inconsistent findings of calbindin-positive cells

Although CB and SST are often co-expressed within the superficial layers of the DLPFC (Gonzalez-Albo et al., 2001; Zaitsev et al., 2009), given the historical difficulty of identifying SST interneurons in schizophrenia (Dienel et al., 2023b), CB has often been used as an independent interneuron marker. However, not all SST-positive neurons are CB-positive (Ang and Shul, 1995; Suarez-Sola et al., 2009), meaning that CB-only investigations likely assess a population different than those identified solely with SST. Therefore, we thought it apt to include studies investigating CB-identified interneurons in a separate section. Indeed, findings of CB-positive interneurons in schizophrenia cortex differ from those of SST findings. In studies of CB-positive interneurons in schizophrenia, mRNA expression of CB within the DLPFC of individuals with schizophrenia was higher than that of matched comparison subjects (Fung et al., 2010). Assessments of CB density in schizophrenia have been varied. Three reports of CB density identified by immunohistochemistry within the DLPFC of individuals with schizophrenia have reported a higher number of neurons within layers 3 and 5/6 (Daviss and Lewis, 1995), unaltered density of CB neurons within any layer (Tooney and Chahl, 2004), and fewer medium-size CB neurons within layer 2 (Sakai et al., 2008). Investigations of other regions and/or disorders have not added clarity. The density of CB interneurons of any size identified by *in situ* hybridization in the anterior cingulate cortex did not differ in schizophrenia or BPD (Woo et al., 2008), whereas the density of large-size CB neurons within DLPFC layer 2 was higher in BPD (Sakai et al., 2008) but lower in MDD DLPFC (Rajkowska et al., 2007). If the inconsistency of the aforementioned results cannot be attributed to methodology or cohort, it may suggest unique subpopulations of CB cells by region, layer, and neuronal size.

## Limited but consistent findings of vasoactive intestinal polypeptide neurons in schizophrenia

Approximately 80% of CR neurons also co-express VIP, and roughly 80% of VIP neurons contain CR (Gabbott and Bacon, 1997). VIP interneurons predominantly target the dendrites of PV and SST neurons (del Río and DeFelipe, 1997b; Rajkowska et al., 2007; Melchitzky and Lewis, 2008; Donato et al., 2013; Pfeffer et al., 2013; Pi et al., 2013), but also synapse onto the dendrites and pyramidal cell somata. By providing inhibition onto inhibitory PV and SST neurons, VIP interneurons mediate disinhibitory control of local pyramidal neurons, resulting in an upregulation of local excitatory activity (Pi et al., 2013). Therefore, VIP neurons play a crucial role in modulating the microcircuitry associated with WM capability. Despite their importance, VIP neurons have been infrequently studied in the postmortem PFC of schizophrenia subjects. However, findings of this neuronal type have been consistent in the illness, with reports of lower PFC VIP mRNA and protein (Gabriel et al., 1996; Fung et al., 2010; Fung et al., 2014). Furthermore, Gabriel et al. (1996) reported less VIP protein expression across multiple cortical regions involved in the visuospatial WM network. Lastly, these findings appear to be consistent with the singular study that has assessed VIP protein in BPD PFC (Fung et al., 2014), suggesting a potentially shared VIP neuron pathology between these two types of serious mental illness, but further work is needed.

## Alterations of neuropeptide Y in cortical gray and white matter

NPY is often co-expressed alongside one of the calcium-binding proteins, and thus can be utilized to identify a particular subset of cortical interneuron. NPY-expressing cells are primarily localized in cortical layers 2/3 and 5/6 (Kuljis and Rakic, 1989). Relatively few, and virtually none, of these cells can be observed in layer 1 and layer 4, respectively (Kuljis and Rakic, 1989). However, NPY-positive cells are highly prevalent within the superficial white matter (Kuljis and Rakic, 1989). In schizophrenia, investigations of NPY expression within postmortem PFC have been varied. Indeed, many reports indicate lower mRNA and protein expression of this neuropeptide within PFC gray matter (Gabriel et al., 1996; Kuromitsu et al., 2001; Hashimoto et al., 2008; Mellios et al., 2009; Fung et al., 2010), although others report no change (Caberlotto and Hurd, 1999; Morris et al., 2009).

Investigations of NPY interneurons within superficial white matter have been limited, but report lower NPY mRNA within the DLPFC of individuals with schizoaffective disorder, but not a separate cohort of subjects with schizophrenia (Morris et al., 2009), suggesting NPY cells might be more perturbed in affective disorders. Indeed, the relatively few studies that have investigated this interneuron type in BPD or MDD PFC have reported lower levels of NPY mRNA expression in BPD, but not MDD (Caberlotto and Hurd, 1999; Kuromitsu et al., 2001), suggesting even further distinction of the NPY interneuron subtype within mood disorder pathology.

## Differential expression of cholecystokinin interneurons in schizophrenia prefrontal cortex

In primate neocortex, CCK-positive interneuron somata are predominately observed in layers 2-superficial 3, whereas their axon terminals primarily innervate laminae 2, 4, and 6 (Oeth and Lewis, 1993; Eggan et al., 2010). These neurons, like PVBCs, target the perisomatic region of local pyramidal neurons and tune their firing rate. However, unlike PVBCs, CCK interneurons are suspected to tune cellular firing at a theta, not gamma, frequency (Traub et al., 1996; Klausberger et al., 2005; Klausberger and Somogyi, 2008), and therefore may be less directly involved in WM capability. Although a subpopulation of CCK-positive cells can be defined by its co-expression of other proteins such as cannabinoid 1 receptor (Eggan et al., 2010), for the purpose of this review we have limited discussion to studies using CCK as its primary identifying marker.

In schizophrenia PFC, lower levels of CCK transcript have been observed (Hashimoto et al., 2008; Fung et al., 2010). Although localized to the same laminae as findings of GAD67 deficits in schizophrenia, assessment of GAD67 within CCK cells in schizophrenia has yet to be conducted. However, investigation of GAD67 and GAD65 within cortical CCK-positive axon terminals indicates that CCK-positive cells may predominantly express GAD65 instead of GAD67 (Fish et al., 2011), and therefore may not contribute to the GAD67 deficit observed in the disease.

Investigations of CCK in BPD or MDD have been extremely limited. Indeed, zero studies were identified in BPD, and only two were identified with potential implications for MDD (Perry et al., 1981; Barde et al., 2024). However, the most recent study conducted by Barde et al. (2024) investigated MDD subjects who died by suicide, and reported elevated activity of the CCK systems in PFC, although these findings may be unique to those who completed suicide (Yin et al., 2016; Dowling et al., 2023).

## GABA in presynaptic boutons in schizophrenia prefrontal cortex

Although difficult to assess directly in human postmortem tissue, measuring levels of mRNA transcripts and their cognate proteins that regulate GABA in presynaptic terminals can serve as an adequate proxy of the strength of GABAergic neurotransmission (Schoonover et al., 2020). Indeed, synaptic GABA is synthesized by the 67 and 65 kDA isoforms of the enzyme glutamic acid decarboxylase, known as GAD67 and GAD65, respectively. However, GAD67 is the enzyme predominantly responsible for the GABA synthesis load (Asada et al., 1997), whereas GAD65 appears to be active only during periods of high neuronal activity (Patel et al., 2006). In response to homeostatic signals of excessive network activity (Erickson et al., 2006; Hartmann et al., 2008), the strength of GABAergic neurotransmission can be upregulated by the increased accumulation of GABA into synaptic vesicles, mediated by vGAT. Lastly, the clearance of GABA from the synaptic cleft is largely regulated by GAT1, responsible for GABA neurotransmitter reuptake at the presynaptic terminal. Furthermore, elevation of synaptic GABA reuptake via GAT1 regulates inhibitory postsynaptic potentials of perisomatic GABA inhibition and prevents GABA spillover at both perisomatic and dendritic-targeting GABA inputs (Gonzalez-Burgos et al., 2009). Together, levels of these three presynaptic markers of GABA neurotransmission in the postmortem brain can provide insight into the integrity of GABAergic signaling in schizophrenia.

In schizophrenia, studies reporting prefrontal GAD67 deficits across multiple methodologies, anatomical levels of resolution, and cell types have been reported (Akbarian et al., 1995; Mirnics et al., 2000; Volk et al., 2000; Vawter et al., 2002; Hashimoto et al., 2005; Straub et al., 2007; Hashimoto et al., 2008; Duncan et al., 2010; Curley et al., 2011; Kimoto et al., 2014; Tao et al., 2018; Lanz et al., 2019; Enwright and Lewis, 2021). For example, two *in situ* hybridization studies revealed a GAD67 mRNA deficit across PFC layers 2–5, with pronounced deficits in layers 3–4 (Akbarian et al., 1995; Volk et al., 2000), and GAD67 protein levels measured by Western blot and immunofluorescence were also reported to be lower in the DLPFC of individuals with schizophrenia (Curley et al., 2011). In contrast, GAD65 mRNA and protein levels have been reported to be unaltered in PFC total gray matter homogenate in schizophrenia (Glausier et al., 2015). However, a recent study investigating levels of GAD67 and GAD65 mRNA in superficial (layers 2/superficial 3) and deep (deep layer 6/superficial white matter) zones of the DLPFC in schizophrenia found zone-specific alterations of both transcripts in subjects with schizophrenia, reporting alterations of GAD67 and GAD65 mRNA in the superficial, but not the deep, zone (Dowling et al., 2023). Together, these data suggest that potential alterations of GAD65 may be limited to specific cellular populations that are obfuscated during measures of broader whole tissue studies. Indeed, when examining GAD67 protein levels within vGAT-positive boutons of the prefrontal cortex, GAD67 levels are significantly lower in the disease (Rocco et al., 2016a), suggesting that GABA production is reduced in a subset of prefrontal boutons in schizophrenia.

In contrast to the relatively robust findings of alterations of GAD67 in schizophrenia DLPFC, vGAT and GAT1 mRNA levels appear to be unaltered or only modestly lower in total gray matter homogenates of PFC (Fung et al., 2011; Hoftman et al., 2015) or PFC layer 3 samples (Hoftman et al., 2018). Furthermore, no significant alterations of vGAT protein levels within prefrontal synaptic boutons, nor the number of vGAT-positive boutons, have been observed (Rocco et al., 2016a). However, these findings may be a result of a lack of cellular-specific investigation. Indeed, it has been suggested that a subset of neurons may be more severely affected in the disease. Volk et al. (2000) reported a ∼25% deficit in the density of GAT1 mRNA-positive neurons in schizophrenia, indicating cellular loss. However, recent advancements of *in situ* hybridization methodology may now allow detection of cells with a previously undetectable GAT1 mRNA level via older methods (Dienel et al., 2023b). Thus, investigation of GAT1 mRNA-positive neuronal density should be conducted utilizing new methodology.

Although studies of presynaptic GABAergic transcripts and proteins within the postmortem PFC in schizophrenia are relatively few given the importance of the system in cognition, investigations of the GABAergic system are even more sparse in mood disorders with similar cognitive impairments as schizophrenia. Indeed, only two studies have been conducted to date investigating GABA synthesis within the postmortem PFC from individuals with schizoaffective disorder (Glausier et al., 2015), BPD (Dowling et al., 2023), or MDD (Dowling et al., 2023). Both GAD65 mRNA and protein in PFC total gray matter may be lower in those with schizoaffective disorder (Glausier et al., 2015). In contrast, Dowling et al. (2023) examined GAD67 and GAD65 mRNA levels in superficial (layers 2/superficial 3) and deep (deep layer 6/white matter) zones of the DLPFC in BPD and MDD subjects, and reported no significant alterations of either transcript, regardless of laminar zone or diagnosis.

## Postsynaptic GABAergic alterations in schizophrenia

In addition to deficits of presynaptic GABAergic function in the interneurons of the DLPFC microcircuitry, findings of postsynaptic alterations in apposition to inhibitory inputs in schizophrenia have been reported (Mohler, 2006; Glausier et al., 2010; Beneyto et al., 2011; Fish et al., 2018). Both the α1 subunit and the β2 subunit of the GABA_A_ receptor are lower in schizophrenia DLPFC layers 3–4 (Beneyto et al., 2011). These subunits colocalize with the α1 subunit at PVBC inputs (Mohler, 2006). Furthermore, PVBC, but not PV ChC, terminals are highest in density within PFC layer 4 (Fish et al., 2018). Therefore, given that the local layer 3 pyramidal neurons likely to receive these inhibitory inputs also exhibit lower levels of α1 mRNA (Glausier et al., 2010), the data together suggest a dampening of the inhibitory signal received by pyramidal neurons from PVBCs.

Additionally, the postsynapse of ChC inputs have been reported to be altered in schizophrenia. As previously mentioned, ChCs are predominantly located within PFC layer 2 (Fish et al., 2018) and exhibit α2-enriched GABA_A_ receptors postsynaptic to their inputs (Nusser et al., 1996; Loup et al., 1998). Within this cortical layer enriched in ChCs (Fish et al., 2018), the α2 mRNA expression and density of α2-positive cartridges have been reported to be upregulated in the illness, suggesting distinct alterations unique to PV interneuron subtype.

Alterations of postsynaptic SST inputs, although infrequently studied, have been reported in a laminar and cell-type specific manner. Indeed, lower levels of the α5 subunit of the GABA_A_ receptor, often found in opposition to SST inputs (Klausberger et al., 2002; Silberberg and Markram, 2007), have been reported to be lower in DLPFC layer 4 (Beneyto et al., 2011). Such an alteration likely reflects abnormalities of the α5 subunit in the PV cells predominantly localized to layer 4 within the cortex, although this remains untested. Together, with the well-replicated downregulation of SST in schizophrenia, these data indicate lower activity of SST neurotransmission. However, a recent publication investigating key ionotropic receptor subunits across four cortical regions, including the DLPFC, in schizophrenia, did not report alterations of the α5 subunit within total gray matter homogenate of the DLPFC, but did report deficits of the subunit in caudal cortical regions (Schoonover et al., 2024), suggesting there could be region-specific alterations in the illness.

Assessment of postsynaptic GABA_A_ receptor subunits in BPD or MDD have primarily been conducted within total gray matter or the somal body of pyramidal neurons in DLPFC, and not at the level of synaptic boutons. However, higher expression of the α1 subunit but lower expression of the β1 subunit within the DLPFC total gray matter has been reported in BPD (Fatemi et al., 2017). In contrast, when assessed at the level of pyramidal neuron soma, a second study reports unaltered protein levels of the α1 subunit, but elevated β2/3 protein levels, in BPD (Ishikawa et al., 2004). Lastly, a third study investigating β2 mRNA levels within total gray matter of the anterior cingulate cortex and DLPFC in elderly non-suicidal MDD patients reported lower expression within the anterior cingulate cortex, but not the DLPFC (Zhao et al., 2012).

## Cortical interneuron pathology in other regions: implications for working memory

Although we have focused on interneuron pathology within human postmortem DLPFC as it pertains to WM impairments in schizophrenia, this does not suggest that there are no alterations within other regions that affect WM capability in the illness. Indeed, the inhibitory neurons within the deep cortical zone of the PFC are known to innervate pyramidal neurons that, in turn, are reciprocally connected to a variety of thalamic nuclei (Preuss and Goldman-Rakic, 1987), and provide inhibition to several other brain regions (Tomioka et al., 2005; Tomioka and Rockland, 2007; Tamamaki and Tomioka, 2010; Tomioka et al., 2015; Tremblay et al., 2016; Lim et al., 2018). Indeed, the thalamus, and particularly the mediodorsal nucleus of the thalamus (MD), has been implicated as crucially involved in the WM network (Niendam et al., 2012; Bolkan et al., 2017; Murray and Anticevic, 2017). The MD, like the PFC, exhibits sustained WM delay-related activity (Watanabe and Funahashi, 2012), and lesions to the MD in humans result in significant impairments in cognitive functions, particularly executive abilities and memory (Dagenbach et al., 2001; Kubat-Silman et al., 2002; Van der Werf et al., 2003).

Although the full complexity of PFC-thalamic connectivity in schizophrenia is beyond the scope of this review, altered signaling from the MD may substantially impact PV interneurons and their functional capability in WM. Indeed, the MD, which has been shown to exhibit reduced connectivity in patients with schizophrenia (Welsh et al., 2010; Woodward et al., 2012; Anticevic et al., 2014a; Anticevic et al., 2014b; Klingner et al., 2014; Tu et al., 2015; Wang et al., 2015; Lerman-Sinkoff and Barch, 2016) and to a lesser extent BPD (Anticevic et al., 2014a; Anticevic et al., 2014b; Woodward and Heckers, 2016), targets several types of interneurons as well as pyramidal neurons within cortical layers 1, 2–3, and 5 (Ferguson and Gao, 2018a; Lewis et al., 2022). Thus, reduced input from the MD to the PFC is likely to result in abnormal excitatory/inhibitory balance within the cortical microcircuitry associated with WM capability. Indeed, the activity of MD neurons is correlated with the spiking of PV interneurons in the medial PFC in mice (Schmitt et al., 2017; Ferguson and Gao, 2018b), indicating that a dampening of the MD activity selectively reduces inhibitory currents onto pyramidal neurons (Ferguson and Gao, 2018b), similar to the findings observed in postmortem tissue as previously discussed. However, postmortem assessments of MD cell counts and volume in schizophrenia have been inconsistent (Heckers, 1997; Pakkenberg et al., 2009; Dorph-Petersen and Lewis, 2017), although similar investigations within the pulvinar nuclei of the posterior thalamus suggest reduced pulvinar volume and cellular density (Dorph-Petersen and Lewis, 2017). Thus, further study is needed of the MD thalamic nuclei in schizophrenia postmortem tissue to identify the neural substrate of the observed *in vivo* MD alterations in the illness.

Taken together, these data indicate that WM capability is subserved by a multi-node network involving both cortical and subcortical regions, and investigations of PFC-only GABAergic neurotransmission likely represent only a part of the larger circuitry associated with WM.

## Challenges in measuring GABA levels directly

This review has largely focused on alterations in molecular and neural circuit properties of GABAergic interneuron subtypes in nonhuman primate or human postmortem PFC from individuals with schizophrenia, which have been essential to our understanding of schizophrenia pathobiology. To continue to uncover the mechanistic mysteries underlying this illness for the creation of better pharmacotherapies, a large portion of research in this field has made use of preclinical techniques and model systems (Canetta et al., 2016; Kaneko et al., 2017; Smith et al., 2019; Arias et al., 2022). Preclinically, physiological techniques like electrophysiology combined with optogenetics have been widely used to study the electrical and physiological properties of cortical interneurons, both *in vivo* and *in vitro*, to electrically stimulate and record the transmission of neuronal signals (Sohal et al., 2009; Anastasiades et al., 2021; Maksymetz et al., 2021). The addition of pharmacological and molecular approaches allowed scientists the ability to decipher extracellular, intercellular, and intracellular signals within the brain to decipher the role of GABAergic interneurons, GABA receptors, ion channels, and certain neurotransmitters in specific neural circuits and behaviors associated with schizophrenia. For example, ketamine has been used to illustrate a dose-dependent effect on PV-positive interneuron physiology in a GAD1-knockdown model of schizophrenia resulting in molecular, physiological, and behavioral changes in mouse models (Brown et al., 2015). Thus, although using electrophysiology combined with pharmacology are classical and established methods of scientific investigation, we still rely heavily on these techniques to be able to infer the activity of neurotransmitters like GABA in preclinical models of aspects of schizophrenia.

Nevertheless, the aforementioned techniques do not directly measure GABA levels, which would be ideal for preclinical schizophrenia research based on the involvement of the GABA system in its underlying neural circuit processes. To guide future preclinical schizophrenia research involving GABA, there is a need for assessment of GABA levels that considers its multidimensional effects. Therefore, the utilization of genetically encoded fluorescent biosensors combined with pharmacology and physiological techniques allows for *in vivo* and potentially *ex vivo* optical assessment of pharmacological manipulation of GABA transients, providing novel understanding of the physiological mechanisms behind the role of GABA in schizophrenia pathology (Marvin et al., 2019; Guo et al., 2021).

In clinical research, the use of different types of electroencephalography (EEG) combined with neuroimaging tools is becoming more widespread in the investigation of GABAergic deficits associated with cognitive symptoms underlying schizophrenia pathophysiology. In relation to WM, several studies have used transcranial magnetic stimulation with EEG (TMS-EEG) to show connections between GABAergic neurotransmission and neural oscillations (Daskalakis et al., 2006; McClintock et al., 2011; Lett et al., 2014). In healthy subjects, single and paired-pulse variations of long interval cortical inhibition (LICI) in the DLPFC have been used with TMS-EEG to implicate GABAergic neurotransmission with performance in WM tasks (Daskalakis et al., 2008; Rogasch et al., 2015). Additionally, LICI and TMS-EEG have been applied in clinical studies of schizophrenia patients and healthy controls to implicate the contribution of GAD67 gene *GAD1* in WM deficits associated with schizophrenia (Lett et al., 2016). Importantly, these studies do not directly assess the relationship between components of GABAergic neurotransmission with WM deficits in patients with schizophrenia specifically but can be used to infer neurophysiological processes for the development and testing of better treatments (Lett et al., 2016).

## Sex differences remain understudied

Sex differences were rarely considered in the science and medicine until the last century (Yoon et al., 2014). Males were commonly used as a baseline of human body function, with hormones as a major perceived difference in females. Additionally, in the rare instances where both male and female subjects were assessed, the data was often not separated and analyzed by sex. We now know that all hormones, even those historically associated with one sex, are prevalent in both sexes and have large effects on the body and emotional processes of the brain (Lauretta et al., 2018; Miguel-Aliaga, 2022). In more modern times, sex differences have been identified in many mood disorders such as MDD and BPD (Douillard-Guilloux et al., 2017; Girgenti et al., 2019). For example, MDD was reported to have sex-specific differences in the number of SST interneurons, where fewer SST neurons were present in females compared to males, suggesting there is likely a neural substrate to the sex differences in the diagnosis of MDD (Douillard-Guilloux et al., 2017; Girgenti et al., 2019). However, even with the advancements made in expanding research into sex differences in serious mental illness, much progress has yet to be made.

After examining the relevant literature previously cited within this review, we found that approximately half of the reports investigated sex as a variable. Of those, approximately 20% reported sex-specific differences that were often complex (relying upon examination of specific cortical layer versus total gray matter) or no longer statistically significant following correction for multiple comparisons. Indeed, subunits of the GABA_A_ receptor did not differ overall by sex in schizophrenia when compared to those unaffected by the disorder but did have higher expression of the GABA_A_ receptor subunit α2 mRNA within the DLPFC layer 2 in males versus female individuals diagnosed with schizophrenia (Beneyto et al., 2011). Furthermore, GABA_A_ receptor subunit α2 mRNA expression within layer 2 was higher in male subjects with schizophrenia than in male unaffected comparison subjects (Beneyto et al., 2011). Additionally, women expressed lower GAD67 and GABA_A_ receptor subunit α4 mRNA (Duncan et al., 2010), but higher SST and lower PV mRNA (Mellios et al., 2009). Significant findings of GABA_B_ receptor subunit protein expression by sex did not withstand correction for multiple comparisons in BPD (Fatemi et al., 2017).

It is important to note that in many studies, including some but not all examined here, subject cohorts are not well-powered enough to adequately assess the effect of sex as a biological variable, often having fewer than 50% female representation. It is crucial to investigate the possibility of sex differences in schizophrenia, given that close to half of patients with this disorder are female (McGrath et al., 2008). Expanding cohorts to include both sexes, comparing data based on sex, and investigating the effect of hormones would greatly improve our current understanding of schizophrenia and its underlying altered neural circuit mechanisms.

## Conclusion

In summary, research into cortical interneurons in schizophrenia reveals a complex pattern of alterations, particularly in the DLPFC. PV interneurons, critical for local inhibitory control, show consistent deficits in mRNA and protein levels, with evidence suggesting reduced functionality rather than a reduction in cellular density. Notably, deficits are observed specifically in PVBCs, but not necessarily in ChCs, indicating subtype-specific effects. In contrast, CR interneurons appear largely unaffected, although regional and methodological variations exist. SST interneurons display robust downregulation of mRNA levels, suggesting impaired inhibitory signaling; however, this does not correspond to a reduction in cell density. Findings related to other interneuron types, such as CB and VIP, are more variable and less consistent, with some reports suggesting elevated or unchanged levels depending on the specific study and methodology. NPY and CCK interneurons also present mixed results, with some evidence of reduced expression in schizophrenia. Finally, the overall GABAergic system shows disrupted function, including reduced GAD67 levels and altered postsynaptic receptor subunit expression. These findings highlight the nuanced and region-specific nature of interneuron pathology in schizophrenia, which may contribute to the cognitive deficits observed in the disorder.

Lastly, research on interneurons in the PFC of individuals with BPD or MDD reveal inconsistent findings. Studies on PV, CR, and SST cells show mixed results, with some evidence of deficits in either BPD or MDD, and cortical layer-specific effects adding complexity. CB cell densities vary by region and layer, showing elevations in BPD and deficits in MDD, although VIP findings suggest possible shared pathology between the two disorders. NPY levels are lower in BPD but not MDD. Research on GAD65 and GAD67 mRNA and protein shows few changes in BPD and MDD. Studies on CCK and GABA_A_ receptor subunits also present limited and variable results. Overall, these inconsistencies highlight the need for more research to better understand the role of these interneurons in cognitive function within affective disorder pathology.
